# Ecological niche modeling and distribution of *Ornithodoros hermsi* associated with tick-borne relapsing fever in western North America

**DOI:** 10.1371/journal.pntd.0006047

**Published:** 2017-10-30

**Authors:** Kylie M. Sage, Tammi L. Johnson, Michael B. Teglas, Nathan C. Nieto, Tom G. Schwan

**Affiliations:** 1 Department of Biological Sciences, Northern Arizona University, Flagstaff, AZ, United States of America; 2 Laboratory of Zoonotic Pathogens, Rocky Mountain Laboratories, National Institute of Allergy and Infectious Diseases, National Institutes of Health, Hamilton, Montana, United States of America; 3 Department of Agriculture, Nutrition, and Veterinary Sciences, University of Nevada, Reno, NV, United States of America; Medical College of Wisconsin, UNITED STATES

## Abstract

Tick-borne relapsing fever in western North America is a zoonosis caused by the spirochete bacterium, *Borrelia hermsii*, which is transmitted by the bite of infected *Ornithodoros hermsi* ticks. The pathogen is maintained in natural cycles involving small rodent hosts such as chipmunks and tree squirrels, as well as the tick vector. In order for these ticks to establish sustained and viable populations, a narrow set of environmental parameters must exist, primarily moderate temperatures and moderate to high amounts of precipitation. Maximum Entropy Species Distribution Modeling (Maxent) was used to predict the species distribution of *O*. *hermsi* and *B*. *hermsii* through time and space based on current climatic trends and future projected climate changes. From this modeling process, we found that the projected current distributions of both the tick and spirochete align with known endemic foci for the disease. Further, global climate models predict a shift in the distribution of suitable habitat for the tick vector to higher elevations. Our predictions are useful for targeting surveillance efforts in areas of high risk in western North America, increasing the efficiency and accuracy of public health investigations and vector control efforts.

## Author summary

The model presented here provides valuable epidemiological information on tick-borne relapsing fever in western North America. The inference gleaned from these models represents areas where human infection with *B*. *hermsii* is likely to occur. The predicted distribution of *O*. *hermsi* and *B*. *hermsii* may allow health officials to decrease human disease burden by implementing targeted surveillance efforts, thus better utilizing resources. The models we created predict the current distribution of *O*. *hermsi* and *B*. *hermsii*, as well as the predicted distribution in 2050 under medium and high greenhouse gas (GHG) concentration trajectories. Understanding how the distribution of the pathogen and its vector expand or contract in response to GHG concentrations is necessary for understanding human risk of infection with this debilitating disease both now and in the future.

## Introduction

Tick-borne relapsing fever (TBRF) is a zoonosis endemic to the Americas, Africa, and Asia, and caused by spirochetes transmitted by soft ticks (Family: Argasidae) in the genus *Ornithodoros* [1]. The disease is caused by a diversity of regionally specific bacterial species in the genus *Borrelia* [2]. Although of low incidence in most endemic regions, TBRF is proposed to be a major cause of fever in Senegal, West Africa, second only to malaria [3, 4]. The clinical disease in humans is characterized by recurring episodes of fever (2–6 episodes) with general symptoms including headache, myalgia, nausea, arthralgia, and vomiting [5, 6]. In North America, three species of TBRF spirochetes are present and each is vectored by a different species of *Ornithodoros*. *Borrelia hermsii*, *Borrelia turicatae*, and *Borrelia parkeri* are transmitted by *Ornithodoros hermsi*, *Ornithodoros turicata*, and *Ornithodoros parkeri*, respectively. Most human cases of TBRF in North America are caused by infection with *B*. *hermsii* [2, 5, 7], which is the focus of our investigation.

In the United States, TBRF was first reported in Colorado in 1915 [8], and was considered endemic there following the collection and identification of *O*. *hermsi* as the primary vector [9]. The geographic distribution of TBRF in western North America is broadly defined by the location of exposure for reported human cases. *Ornithodoros hermsi* has been documented at elevations ranging from less than 3,000 feet to over 8,000 feet in mountainous areas of Colorado, Utah, Idaho, Washington, California, and Montana [10–15]. Human exposures occur most often while sleeping in rustic cabins located in mid to high elevation coniferous forests occupied by tree squirrels (*Tamiasciurus* spp.) and chipmunks (*Tamias* spp.) [1, 5, 16–18]. Recent work demonstrates a greater diversity of small mammal species also serve as hosts for *O*. *hermsi* and *B*. *hermsii* [14].

The geographic range and diversity of potential hosts associated with the enzootic maintenance of *B*. *hermsii* provides a wide distribution across western North America. However most human cases of relapsing fever have originated in a relatively small and highly focal number of locations. For example, from 1990 to 2002 approximately 50% of all human cases in the United States were infected in just 13 counties [19]. Endemic areas with repeated human infection are well documented and include many popular tourist destinations including the North Rim of Grand Canyon National Park (AZ), Estes Park (CO), and several mountain lakes including Lake Coeur D’Alene (ID), Lake Tahoe and Big Bear Lake (CA) and Flathead Lake (MT) [19]. Despite the abundance of potential hosts across the landscape, focal clustering of human cases of TBRF suggests there may be constraints other than the presence or absence of a suitable host for the tick vector. Like other vector-borne diseases, the spatial distribution of TBRF is likely multifactorial and constrained by environmental parameters (biotic habitat and abiotic climate conditions) in addition to host availability and their dispersal, which affect the distribution of *O*. *hermsi*.

The spatial distribution of vector-borne zoonotic pathogens depends heavily on environmental features and of course the presence of both host and vector required for their maintenance in natural foci [20, 21]. The distribution of tick-borne pathogens and the effect of climate on hard ticks (Acari: Ixodidae) has been modeled extensively, however, the effects of climate on soft ticks (Acari: Argasidae) is less well understood, in part due to their cryptic nature and nidicolous lifestyle that make them difficult to find in nature. Hard and soft ticks have vastly different life histories and feeding behaviors, and thus are exposed to different environmental pressures. Hard ticks quest in the open environment for long periods of time to encounter and attach to a host [22]. This life-history strategy means that hard ticks are at risk of desiccation while questing, a process that defines both their survival and phenology, and hence distribution. In contrast, soft ticks do not quest in the habitat, and feed and detach quickly to ensure they remain in or very near to the burrow or nest of the host [23]. Specifically, *O*. *hermsi* ticks feed quickly in all life stages (15–90 minutes), are nocturnal, and thus usually feed when the host is inactive or when people are sleeping [13]. When these ticks drop of their host, they likely remain in the confines of a relatively stable and moderated microclimate [2]. Soft ticks may be less affected by rapidly changing environmental conditions as compared to hard ticks, and therefore may be most influenced by extremes in environmental conditions over the course of their lifetime. Argasid ticks also have cement in the epicuticle, which make them more resistant to desiccation at higher temperatures compared to ixodid ticks [24]. Yet despite these morphological features that enhance survival, there is still a narrow set of environmental parameters that define the physiological threshold required for *Ornithodoros* survival [4, 25]. Additionally, Argasid ticks are long-lived and can survive for many months to years between blood meals, making them both the vector and efficient *de facto* reservoirs for the pathogen [13, 26–28].

Disease ecologists have recently adopted ecological niche modeling (ENM) to predict regions of occurrence and the probability of vector and pathogen shifts in their distribution. ENM is frequently used by ecologists and disease ecologists to better understand species distributions. One program, Maxent, consistently outperforms other ENM models [29, 30] and was developed specifically for data with low sample-sizes of presence-only locations [31, 32]. Initially designed to evaluate the potential distribution of endangered and threatened species, Maxent has been used extensively to model the distribution of numerous arthropods, including soft ticks that vector important disease-causing pathogens [33–35]. The specificity of suitable living conditions for ticks make *O*. *hermsi* and its specific spirochete *B*. *hermsii* prime candidates for ecological niche modeling. The aim of this paper is to use Maxent modeling to describe the current distribution of *O*. *hermsi* and *B*. *hermsii* using documented occurrences of both the tick and spirochete. Further, we apply environmental constraints that predict the effects of various greenhouse gas (GHG) concentration trajectories on their distribution in the year 2050.

## Methods

### Tick and *Borrelia* occurrence data

We used georeferenced presence points for specific locations that included three types of data: 1) human TBRF cases caused by *B*. *hermsii*, 2) *O*. *hermsi* ticks and 3) rodents infected with *B*. *hermsii* based on bacterial isolation or qPCR assays, or positive for anti-*B*. *hermsii* antibodies. Presence locations were obtained from the published literature (when detailed locations were included), as well as a series of samples from this study and personal communications (TG Schwan, NC Nieto and MB Teglas, and KL Gage (see S1 Table). These sites included several popular vacation destination lakes in Washington, Idaho, California, Montana, and British Columbia, as well as several other locations in the Cascade, Sierra Nevada, San Bernardino and Rocky Mountain ranges [18, 36]. *Ornithodoros hermsi* has been documented in many of these areas [10–15, 37–41]. Since *B*. *hermsii* is vector-specific, we are confident that confirmed human cases caused by *B*. *hermsii* represented areas where *O*. *hermsi* was present even if no tick specimens were collected.

### Environmental data

We sought to identify climate variables that are conducive to the persistence of the tick vector *O*. *hermsi* and its specific pathogen *B*. *hermsii*. Climatic variables and elevation were obtained from WorldClim [42], a freely available and widely used dataset of global climate layers, at a spatial resolution of 30 arc-seconds (~1 km; http://worldclim.org). These data represent an interpolation of average monthly climate data recorded at weather stations throughout the region. We chose to eliminate correlated variables to decrease model complexity and increase the interpretability of model output [30]. We identified highly correlated variables (Pearson’s r ≥ ǀ0. 75ǀ) using the Band Collection Statistics Tool in ArcMap (v 10.3, ESRI, Redlands, California, USA), which calculates the Pearson’s correlation coefficient (r) between all pairs of climate variables and elevation. Redundant variables were reduced to a single variable that best represented the most extreme environmental effect of cold and humidity tolerance for ticks, and only these variables were carried forward for model creation and validation. For example, we chose minimum or maximum monthly or quarterly variables over mean or annual variables. Extremes in environmental conditions were chosen due to the life cycle of *O*. *hermsi*, which spends most of its life off the host and sheltered in the relatively stable microclimate of the host’s nest or burrow. Thus, these ticks are most likely affected by extreme climate events that affect the microclimate of the ticks’ immediate environment.

Climate models based on the Intergovernmental Panel on Climate Change 5^th^ Assessment (IPCC5) were also downloaded at a resolution of 30 arc-seconds (~1 km) from WorldClim (www.worldclim.org). We chose three global climate models (GCMs)—ACCESS1-0, HadGEM2-ES, and CCSM4—that have been shown to have better agreement with observations than older models [43]. Two representative concentration pathways (RCP 4.5 and RCP 8.5) were chosen and represent predicted GHG concentration trajectories adopted by IPCC5 and commonly used in the construction of GCMs [44]. Two RCPs were chosen in order to represent medium gas concentrations (RCP 4.5) and high concentration potentials (RCP 8.5).

### Ecological niche modeling

**Fig 1:**
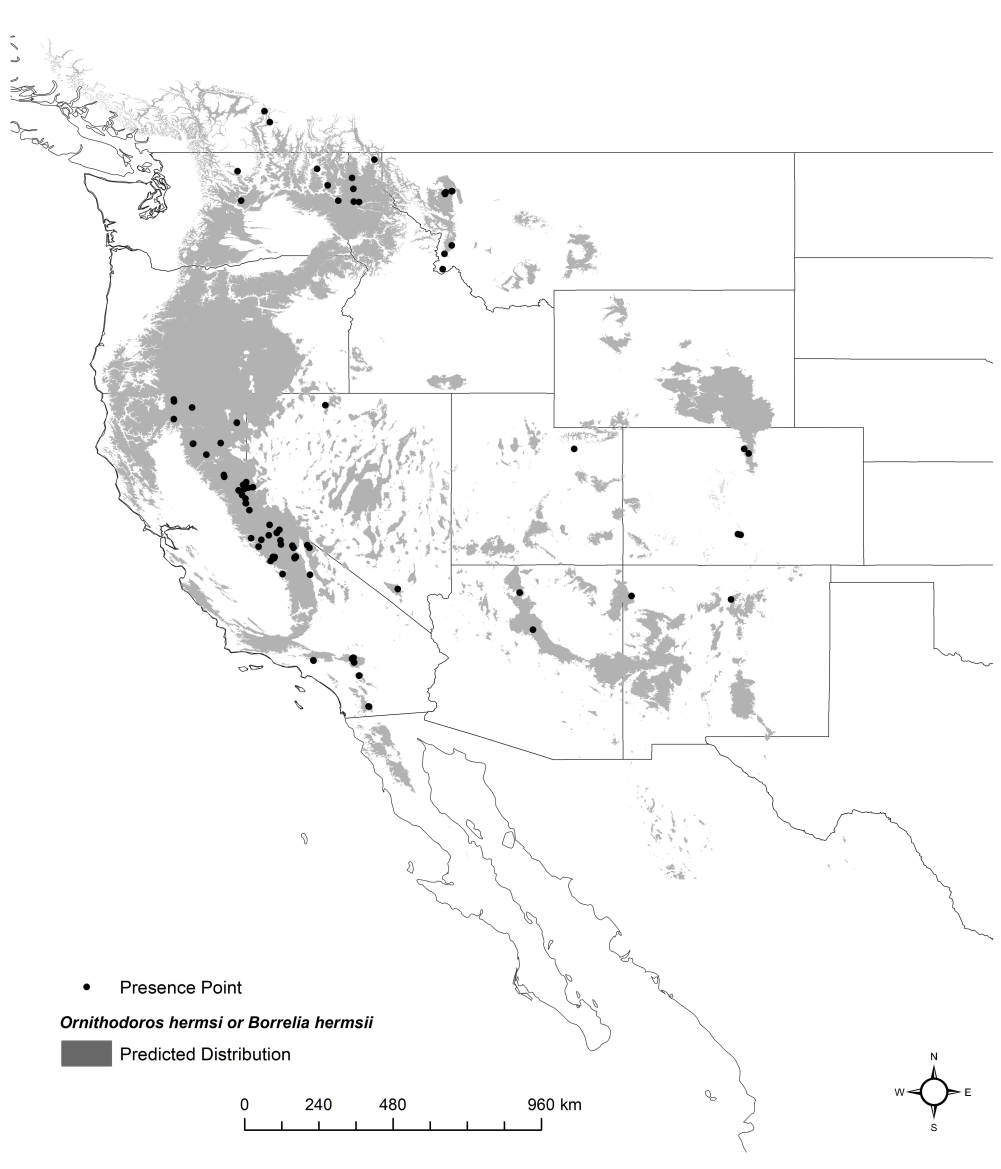
Georeferenced locations of 96 presence points (black dots) and the predicted distribution (gray shading) of *Ornithodoros hermsi* and *Borrelia hermsii* in western North America under current climate conditions. Created in ArcMap (10.2, ESRI, Redlands, CA; http://desktop.arcgis.com/en/arcmap/) using environmental data from WorldClim version 1.4 (http://www.worldclim.org) [42].

*Ornithodoros hermsi* and *B*. *hermsii* presence data were modeled using Maxent version 3.3.3k (https://biodiversityinformatics.amnh.org/open_source/maxent/). Maxent uses presence-only data in combination with environmental data and background pseudo-absences to predict current and future distributions of a species, based on the principle of maximum entropy [31, 32]. Background points were chosen (default N = 10000) at random from western North America. We limited our area of interest (AOI) to the area shown in Figs 1, 4 and 5 as this encompasses the reported endemic regions of *B*. *hermsii* infection in the US. Maxent identifies the broadest probability distribution that falls within a set of constraints to ensure that the distribution reflects information contained in the presence points and to avoid over fitting of the model [31, 45]. Constraints ensure that the mean of each variable used in the model is close to the mean of the variable over occurrence sites, and a regularization parameter prevents over-fitting to occurrence locations [31].

We first developed a “full model” that included all of the uncorrelated environmental variables (described above) and all default Maxent settings, with the addition of 1,500 iterations and 10 cross-validation replicates. We assessed contribution of each variable to the model in two ways, permutation importance and jackknife tests. Permutation importance was determined by randomly permuting each variable among the presence and background training points and measuring the resulting decrease in training Area Under the Curve (AUC) of the Receiver Operating Characteristic (ROC) curve. ROC curves are commonly used in clinical medicine and were designed as a general method for assessing classification performance, where within a continuous data set, an effective threshold is calculated and numbers above the threshold indicate the occurrence of an event [46]. The AUC is a measure of model performance, independent of any chosen threshold, and in the context of our study, represents the probability that a presence point will be ranked above a randomly chosen background point [31]. Maxent normalizes these values to percentages and a large decrease indicates that the model was heavily reliant on that variable. Jackknife tests evaluate and compare AUC values of the model utilizing all variables, with models created using only a single variable in turn and models leaving out one variable in turn. Examination of jackknife plots reveals which variables are contributing the most unique information to the model. After examining model output from the “full model” we chose to simplify the model by excluding variables that were not contributing to model fit, as described above. Using only those variables that contributed considerably to the “full model” (≥5 permutation importance or ≥5% contribution), we created a “reduced model” to predict the distribution of TBRF.

The “reduced model” included all default Maxent settings with the following modifications: 1500 iterations, 10 replicate (cross-validation) models, and Hinge features. Hinge features are capable of modeling piecewise linear responses to variables and allow for simpler and more succinct approximations of the response to environmental variables. Hinge features improve model performance and smooth the fit to the data, thus simplifying the fitted features [45, 47, 48]. Model performance was assessed using the average AUC_test_ statistic. Additionally, we created average response curves from the 10 model replicates for each variable to explore how the logistic probability of suitability changed as each variable was permuted. To visualize the geographical distribution given by Maxent, we created a binary distribution surface of western North America using the 10^th^ percentile logistic training threshold, which assumes that 10% of the presence data may be prone to errors. This is a conservative estimate often used when presence data are collected over a long time span and derived from multiple sources [49]. To evaluate the effect of climate change on the predicted distribution of suitability, we used the “Projection” option in Maxent. We applied the “reduced model” to climate conditions under three GCMs and two emission scenarios and compared model consensus among GCM models under each RCP and visualized the distribution using the logistic cutoff (described above). We developed the consensus maps by reclassifying each model (that is, all suitable pixels for the first model were given a value of 1, all suitable pixels for the second model were given a value of 10, and all suitable pixels for the third model were given a value of 30). We then used Raster Calculator to “add” the models together to produce a single distribution showing three categories: 1) all areas predicted suitable by one model, 2) all areas predicted suitable by two models, and 3) all areas predicted suitable by all models.

## Results

**Fig 2:**
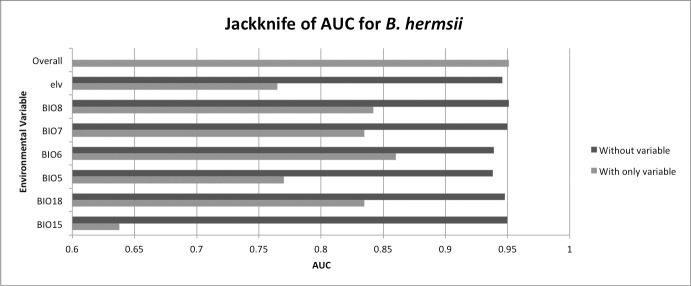
Jackknife analysis of the top environmental predictors used in the model. Jackknife tests of variable importance for 10 replicate models were performed using only single variables (light gray bars) and without each variable (dark gray bars). The corresponding decrease in AUC was measured; large decreases in AUC illustrate model dependence on variables. BIO5 = maximum temperature of the warmest month, BIO6 = minimum temperature of the coldest month, BIO8 = mean temperature of the wettest quarter, BIO7 = annual temperature range, BIO15 = precipitation seasonality, BIO18 = precipitation of the warmest quarter, elv = elevation.

We incorporated 96 georeferenced locations of 1) human TBRF cases infected with *B*. *hermsii*, 2) the presence of *O*. *hermsi*, and 3) rodents infected or previously infected with *B*. *hermsii*. These data were incorporated into a presence-only ENM program to predict the distribution of *O*. *hermsi* in western North America and to assess the effect of environmental variables on the given distribution (Fig 1). In total, seven environmental predictors contributed to model fit, and their importance was conserved across training, testing, and AUC regularization gain throughout all ten replicate model runs (Table 1). The mean AUC_test_ for the 10 replicate models was 0.95 (s.d. = 0.02). The average 10^th^ percentile logistic training threshold of 0.14 was used as the cutoff to create a binary map of the potential distribution (Fig 1). Three variables contributed had high permutation importance, accounting for 79.6% of the variation in the model (Table 1). Jackknife analysis of variables showed that the minimum temperature of the coldest month, the mean temperature of the wettest quarter, temperature annual range, and the amount of precipitation during the coldest quarter contained the most influential information when used alone in the model (Fig 2). The maximum temperature of the warmest month contained the most unique information that was not captured among other predictors, followed by the minimum temperature of the coldest month (Fig 2).

**Table 1:** Estimates of the relative contribution of elevation and six uncorrelated environmental variables used to model the distribution of *Ornithodoros hermsi* and *Borrelia hermsii* in western North America.

Variable	PC^a^	PI^b^	LSR^c^
Minimum temperature of the coldest month (BIO6)	11.6	46.8	-10°C to -5°C
Maximum temperature of the warmest month (BIO5)	14.9	20.3	24°C to 29°C
Elevation	18.1	12.5	> 1700 m
Precipitation of the warmest quarter (BIO18)	17.3	8.5	25 to 75 mm
Annual temperature range (BIO7)	18.9	8.7	26°C to 36°C
Mean temperature of the wettest quarter (BIO8)	11.8	1.7	-4°C to 4°C
Precipitation Seasonality (Coefficient of Variation) (BIO15)	7.5	1.5	62 to 85

^a^ Percent Contribution

^b^ Permutation Importance

^c^ Logistic Suitability range

**Fig 3:**
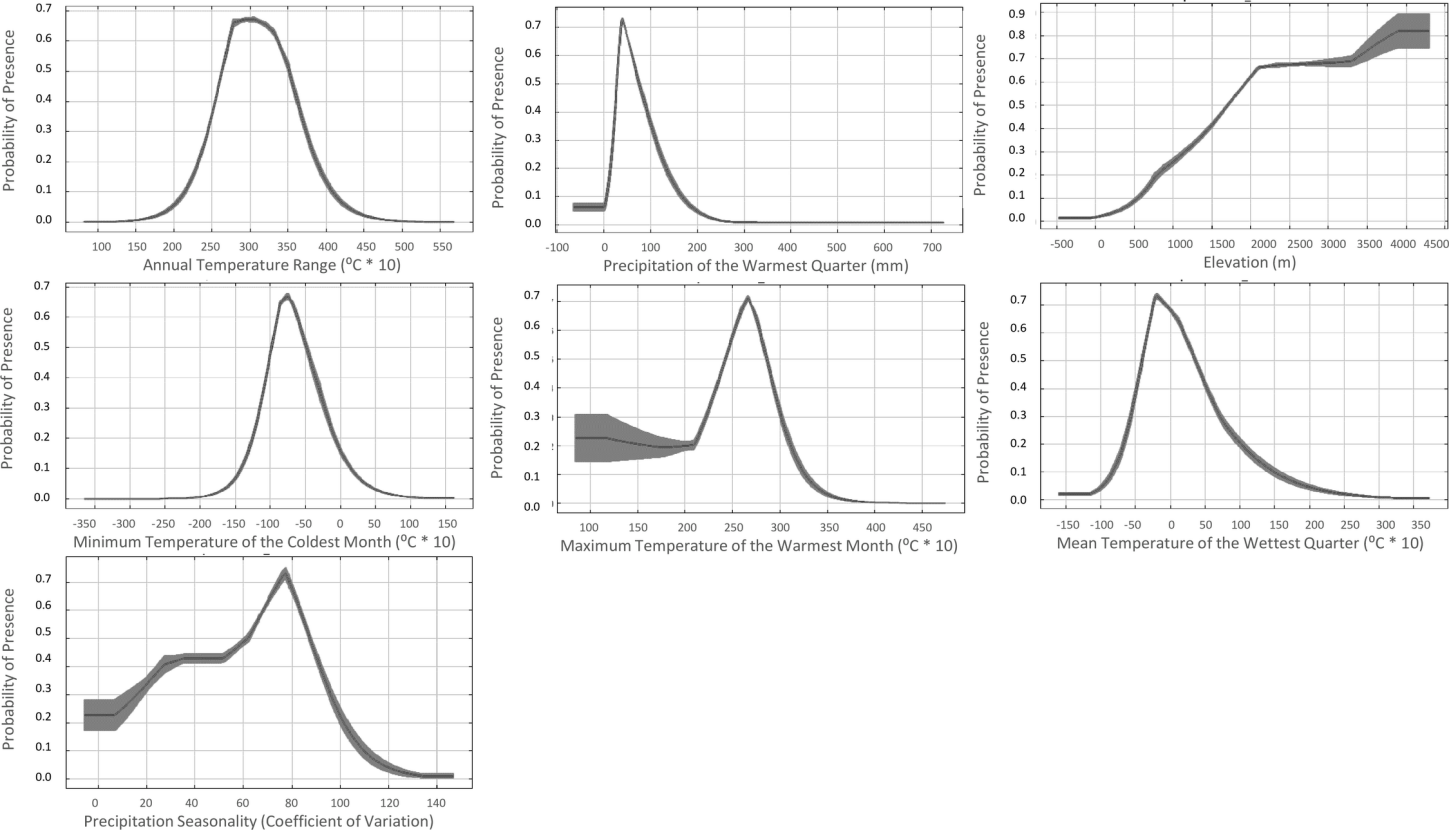
Logistic response curves for the occurrence of *Ornithodoros hermsi* and *Borrelia hermsii* in western North America. The mean (dark gray line) ± one standard deviation (light gray shading) of the 10 replicate Maxent models.

The effect of changing the values of each climate variable on the predicted distribution was examined using variable response curves. The response curves show a narrow range of high suitability for all climate variables while the response curve for elevation shows a steady increase in probability or suitability as elevations increase (Fig 3). The highest probability of suitability is found in regions with moderate temperatures during the wettest quarter of the year (approximately -4°C to 4°C) as well as moderate winter temperatures (approximately -10°C to -5°C) (Fig 3). The highest probabilities of suitability occur at elevations over 1,700 m (Fig 3). The predicted distribution corresponds to areas endemic for TBRF and also correlates with the currently known distribution of *O*. *hermsi* (Fig 1). The distribution encompassed known endemic mountain ranges including the Sierra Nevada and San Bernardino Mountains in California, the Cascade Range in Oregon and Washington, and the Rocky Mountains extending from British Columbia to Mexico (Fig 1). The model also predicted suitable habitat in regions that are not considered endemic for TBRF, including the mountains of northern Baja California, Mexico (Fig 1).

**Fig 4:**
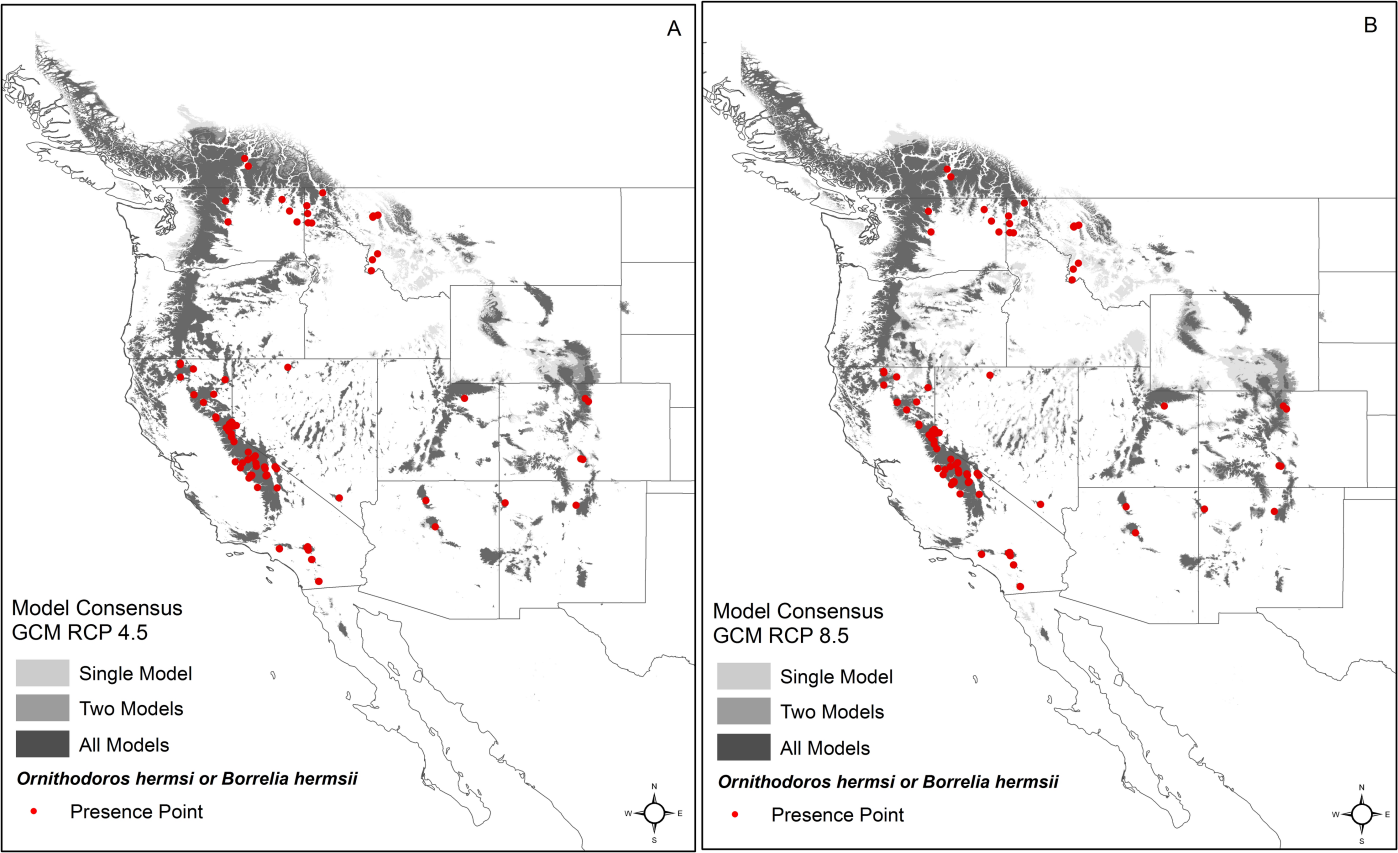
Model consensus for the predicted distribution of *Ornithodoros hermsi* and *Borrelia hermsii* under climate change in 2050. The map shows model consensus among three global climate models (GCMs)—ACCESS1-0, HadGEM2-ES, and CCSM4—and two estimates of greenhouse gas (GHG) concentration trajectories: A) RCP 4.5, a medium estimate of GHG concentrations, and B) RCP 8.5, a high estimate of GHG concentrations. Created in ArcMap (10.2, ESRI, Redlands, CA; http://desktop.arcgis.com/en/arcmap/) using environmental data from WorldClim version 1.4 (http://www.worldclim.org) [42].

**Fig 5:**
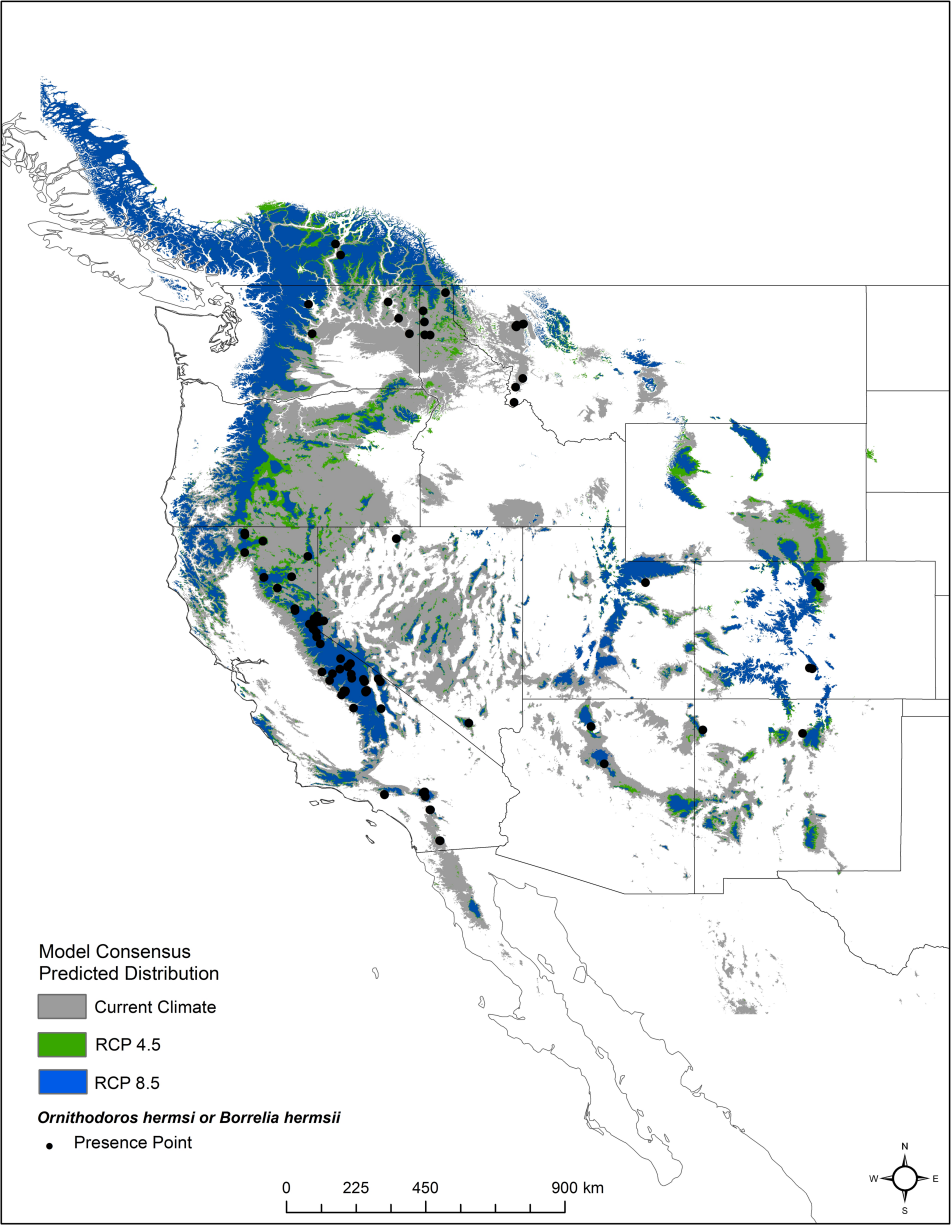
Comparison of current versus future distribution of *Ornithodoros hermsi* and *Borrelia hermsii* predicted by Maxent. The map shows the current predicted distribution in comparison to the consensus of all three global climate models (GCMs)—ACCESS1-0, HadGEM2-ES, and CCSM4—under two scenarios of greenhouse gas (GHG) concentrations, RCP 4.5 and 8.5, representing medium and high estimates of GHG concentrations, respectively. Created in ArcMap (10.2, ESRI, Redlands, CA; http://desktop.arcgis.com/en/arcmap/) using environmental data from WorldClim version 1.4 (http://www.worldclim.org) [42].

**Fig 6:**
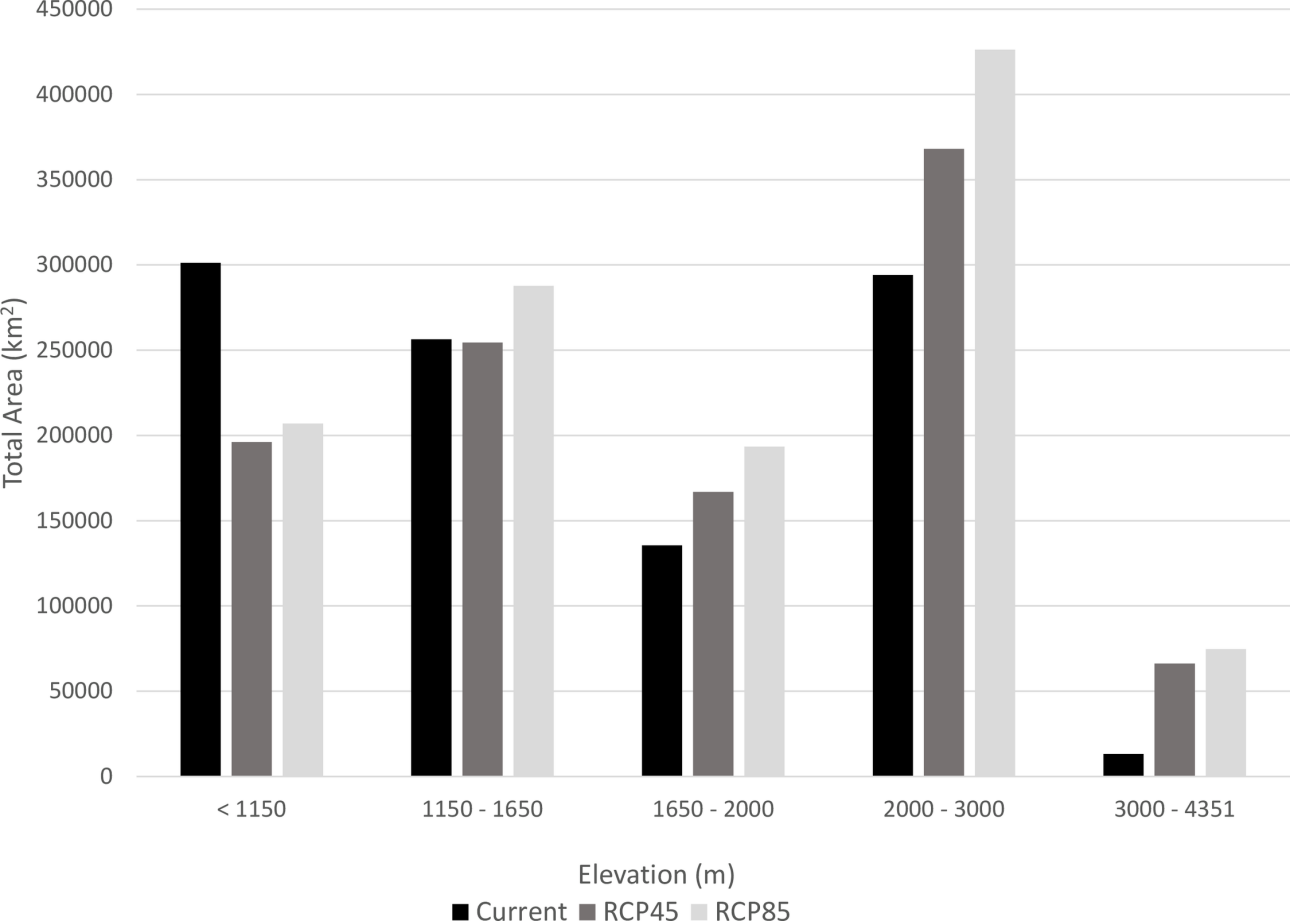
Change in distribution of elevation ranges predicted by each model. Global climate models for 2050 exhibit a shift of suitable climate to higher elevations.

We applied the environmental constraints first identified by the reduced model to climate conditions predicted to occur in 2050 using three GCMs and two GHG concentration pathways (RCP 4.5 and RCP 8.5; Fig 4). Under each RCP scenario, the global mean surface temperature is predicted to increase from 0.9 to 2.0°C under RCP 4.5 and 1.4 to 2.6°C under RCP 8.5 [50]. Two of the most important variables, in addition to elevation, defining the distribution under the current climate were the minimum temperature of the coldest month and the maximum temperature of the warmest month. Under different climate scenarios, the range of suitability for temperature is found at higher elevations. However, the overall amount of area and elevation range predicted as suitable does not change dramatically under predicted climate scenarios (Table 2; Fig 5). Overall, using future climate predictions, a greater percentage of the distribution is predicted to occur at higher elevations (Fig 5). There are notable changes to the predicted distribution in the Cascade Mountains in Washington and Oregon, the Blue Mountains in Oregon, as well as in the Okanagan Highlands in northern Washington and southern British Columbia (Fig 5, Fig 6). Contraction of the distribution is also predicted to occur along some lower ranges, including the Sierra Nevada Mountains. However, expansion is predicted to occur within the Rocky Mountains from southern Wyoming to southern New Mexico, and Utah (Fig 6).

**Table 2:** Total amount of land area predicted as suitable for *Ornithodoros hermsi* and *Borrelia hermsii* in western North America under current climate conditions, as well as global climate modeled under two greenhouse gas (GHG) concentration trajectories for the year 2050. Three global climate models (GCMs)—ACCESS1-0, HadGEM2-ES, and CCSM4—and two GHG concentration pathways, RCP 4.5 and 8.5, representing medium and high estimates of GHG concentrations, respectively, were utilized.

Model	Area Predicted Suitable km^2^ (% total area modeled)
**Current Climate**	1,000,528 (12)
**GCM-RCP 4.5**	
ACCESS1-0	821,216 (10)
CCSM4	893,112 (11)
HadGEM2-ES	829,554 (10)
**GCM-RCP 8.5**	
ACCESS1-0	654,552 (8)
CCSM4	846,651 (10)
HadGEM2-ES	796,332 (10)

## Discussion

The model presented here helps to better define the environmental niche for tick-borne relapsing fever caused by *B*. *hermsii* and its vector *O*. *hermsi* in western North America and for identifying areas of increased risk for human infection. The prediction map created from this model—trained on existing occurrences of *O*. *hermsi* and *B*. *hermsii—*highlights areas with a high probability of tick vector occurrence based on suitable environmental conditions. The Sierra Nevada Mountain Range in California, the Cascade Range and Blue Mountains in Oregon and Washington, the Rocky Mountains in Idaho, Utah, Montana, and Colorado, and the Kaibab Plateau in northern Arizona, are all known endemic sites for TBRF, and the distribution map produced here parallels these areas. This overlap suggests that the model is accurate and correctly identifies regions endemic for TBRF. This model identified geographic areas in which *O*. *hermsi* and *B*. *hermsii* have been identified previously, with the exception of the occurrence of *B*. *hermsii* and *O*. *hermsi* in the northern regions of Baja California, Mexico, a region with no known *B*. *hermsii-*caused TBRF human cases, although other species of *Ornithodoros* do exist [51] (Fig 1). The probability distribution of the model also identified areas where the probability of presence is high, but no cases of relapsing fever have been reported (Fig 1). These areas include a large portion of the Coastal Range in southern Oregon and northern California and smaller but highly suitable regions in northern Baja California, Mexico, the Laramie Mountains,Wyoming, south central Idaho, the Zuni Mountains, New Mexico, and portions of the Uinta and Wasatch Mountains in Utah. Additionally, portions of the Monitor Range, Nevada, were predicted to have suitable habitat. The predictive map produced from our model offers insights into areas where targeted surveillance should be prioritized.

We found that maximum temperature of the warmest month (BIO5), minimum temperature of the coldest month (BIO6), and elevation were most influential for predicting suitability. The logistic response curves demonstrated the narrow range of predicted suitable conditions for the existence of the tick, with many of these curves having defined peaks (Fig 3). This is consistent with previous findings that soft ticks show a strict and narrowly defined tolerance to temperature and humidity for development and activity [52]. Logistic probability distributions indicated that *O*. *hermsi* ticks are semi-cold tolerant, with an optimum minimum temperature during the coldest month and mean temperature of the wettest quarter of approximately -7.5°C. Finally, areas with high predicted probability receive between 25 and 75 mm of precipitation during the wettest month (Fig 3). The probability of suitability also increases with increasing elevation.

The information obtained from the predictive maps of the current distribution of TBRF caused by *B*. *hermsii* was compared to those assembled from the series of future predictions in 2050 with a medium GHG concentration scenario (RCP4.5) and a high GHG concentration scenario (RCP8.5). Global climate models trained on the existing potential distribution showed a relatively stable estimate for the amount of land area that was classified as suitable for *O*. *hermsi*, and therefore *B*. *hermsii*, across western North America. The two emissions scenarios we modeled (RCP 4.5 and RCP 8.5) produced very similar predicted distributions, although the pathway of high concentrations of GHG predicted slightly less overall area (Fig 6). There was a predicted shift in the distribution with suitable areas moving from lower elevation and presumably warmer climates, to climates at higher elevations where conditions may become more suitable (Fig 6). There is potential important habitat gain in the Rocky Mountains of southern British Columbia, Utah, Wyoming, and Colorado and in the Wasatch Range, Utah (Fig 5). Regions of high predicted probability in 2050 were found near Yellowstone National Park, an area encompassed by the Teton and Wind River Mountain ranges, and east in the Big Horn Mountains, Wyoming, and the western front of the Rocky Mountains, Colorado. Climate models for the predicted probability distribution in the year 2050 showed an increase in area predicted at higher elevations (Fig 6) and much of the habitat at lower elevations is predicted to be unsuitable for the tick (Fig 5). In 2050, significant amounts of suitable tick habitats are lost throughout the western United States. A predicted contraction of the suitable habitats occurs throughout the foothills of the Cascade and Sierra Nevada Ranges, and the Rocky Mountains in Montana and Idaho. A considerable amount of *O*. *hermsi* habitat is predicted to be lost in southern California, Baja California, Mexico, central Arizona, and western New Mexico and Nevada (Fig 5).

Interestingly, the contraction of suitable habitat that we see with *O*. *hermsi* and *B*. *hermsii* parallels recent contractions of *Tamias* spp. that have been documented as a result of climate change [53]. For example, the alpine chipmunk *T*. *alpinus* is native in the high Sierra Nevada Mountains in California, and its distribution has noticeably retracted into higher elevations as a result of rising temperatures over the last century [53, 54]. Further, *T*. *palmeri—*endemic to the Spring Mountains in southern Nevada—has predicted constraints to lower slopes, near water sources, and within conifer forests above 2400m, and due to physiological constraints, high temperatures may force this species into higher elevations [55]. Rubidge et al. (2010) found that one chipmunk species, *T*. *senex*, which occupies a low to mid-elevation zone, has become extremely rare in their study area in Yosemite due to a massive range collapse, which may be attributed to warming impacts on vegetation structure. Similar patterns—and even total habitat loss—have been predicted with the red squirrel, *Tamiasciurus hudsonicus*, and other mammalian wildlife populations across the US National Park system [56]. However, it is important to note that not all *Tamias* and *Tamiascurus* species are retracting to higher elevations, or even retracting at all [54].

In the construction of this model, we did not consider any biotic factors, such as vertebrate hosts and their dispersal capability that may influence the potential distribution of the tick and pathogen. The primary rodent hosts for *O*. *hermsi* and thus *B*. *hermsii* in North America include chipmunks (*Tamias* spp.) and tree squirrels (*Tamiasciurus* spp.), however a wider variety of small mammal and bird species likely serve as hosts for *O*. *hermsi* [1, 9, 14, 16, 17]). The geographic range of potential hosts associated with *O*. *hermsi* provides a potential distribution across much of the western United States and southern central British Columbia. In addition to the known importance of rodents as hosts, *O*. *hermsi* has been associated with a variety of wild birds and bats, which may serve as dispersal mechanisms to access previously uninhabited areas [10, 27, 57–61]. Dispersal of *O*. *hermsi* and the potential for infected hosts to disperse *B*. *hermsii* across the landscape is not well understood, however the possibility for aerial dispersal exists for both organisms [14]. Birds are well-known dispersers of *Ixodes* spp. ticks that transmit Lyme disease spirochetes and tick-borne encephalitis virus [62–71]. Moreover, human activities should not be ruled out as potential dispersers of *O*. *hermsi*, as *O*. *hermsi* has been found in sleeping bags and bedding from a cabin [40, 51, 72].

As the global climate warms, the risk of TBRF infection may decline in areas of lower elevation and eventually *B*. *hermsii* transmission may be confined to isolated mountain refugia that maintain suitable climates for the tick. Similar studies have modeled other tick-borne pathogens such as tick-borne encephalitis in Europe, where the tick was reduced to living at higher altitudes because of sensitive climatic and other abiotic suitability ranges [73]. Changes such as this could potentially lead to a noticeable increase of TBRF infections in humans who visit these sites because the probability of tick occurrence is greater, while the potential risk at lower elevations is reduced. Many environmental niche models of vector-borne diseases projected onto future climates show not only a shift in species distribution, but often substantial increases in the amount of suitable habitat. Studies of *Ixodes*-Lyme disease systems in North America and Europe consistently predict a continued expansion of range to higher latitudes [73, 74,70]. The range of leishmaniasis and their sand fly vectors are also predicted to expand in the face of climate change in North America and in Portugal [75, 76]. Similar trends have been predicted in the southern hemisphere where mosquito-borne viruses are expected to expand southward as temperatures rise [77].

Finally, as mentioned previously, two other species of soft ticks in North America, *O*. *parkeri* and *O*. *turicata*, also serve as vectors for relapsing fever *Borrelia* [2]. Modeling the potential distribution of these tick species to determine if there is any environmental overlap in their distributions with *O*. *hermsi* might offer insights for understanding this vector-pathogen specificity. The high correlation of known presence points with areas of high predicted suitability suggest the model presented here is a good representation of the risk for human TBRF. Donaldson *et al*. (2016) modeled the distribution of *O*. *turicata* using Maxent and found that regions of Arizona have a high probability of suitable habitat for this tick, which overlap with regions where *O*. *hermsi* is found. Further, their model also shows low-probability suitable regions for *O*. *turicata* throughout New Mexico and Nevada [34] that have the potential to create further overlap between these two species. As the climate changes, important overlaps in the distribution of these species may change the frequency of human TBRF cases as the potential for tick-host interactions increase.

Spatial models like the one created here have the potential to provide important insights into disease ecology, epidemiology, and the effects of climate change on the distribution of human vector-borne diseases. The results of this model also provide information to researchers investigating the ecology of relapsing fever and aid health care practitioners to achieve a better understanding of where endemic foci may exist. Ultimately, we hope to enhance the recognition of TBRF, which currently is most likely under-diagnosed. Many of the areas with high probability of presence are recreational sites that experience high numbers of human visitation and use. This research will help health care managers in those areas to warn visitors of the potential risks of contracting relapsing fever and what preventative measures should be undertaken to lessen the risk of infection. Visitors to endemic areas who are made aware of the potential to contract TBRF can advise attending physicians of their history of possible exposure that may assist in the diagnosis of tick-borne relapsing fever and appropriate antibiotic therapy.

## Supporting information

S1 Table Occurrence points used in the construction of the model. **One geographic location may represent more than one occurrence point on the map.** ^a^ Indicates where *O*. *hermsi* has also been documented; ^b^ N. Nieto and M. Teglas, *personal communication*; ^c^ Indicates presence of seropositive and PCR positive rodents; ^d^ T. Schwan, *this study*; ^e^ K. Gage, Centers for Disease Control and Prevention, *personal communication*.
(DOCX)
