# Supplementary material for: Ecological niche modeling and distribution of Ornithodoros hermsi associated with tick-borne relapsing fever in western North America
Source: PLoS Negl Trop Dis. 2017 Oct 30;11(10):e0006047. doi: 10.1371/journal.pntd.0006047 (PMC5679642; doi:10.1371/journal.pntd.0006047)
Supplement: S1 Table — One geographic location may represent more than one occurrence point on the map. a Indicates where O. hermsi has also been documented; b N. Nieto and M. Teglas, personal communication; c Indicates presence of seropositive and PCR positive rodents; d T. Schwan, this study; e K. Gage, Centers for Disease Control and Prevention, personal communication. (DOCX) [file pntd.0006047.s001.docx]

S1 Table. Occurrence points used in the construction of the model. One geographic location may represent more than one occurrence point on the map.

| **Location** | **County** | **State/**  **Prov.** | **Latitude/Longitude** |
| --- | --- | --- | --- |
| North Rim Grand Canyon National Park^[37]a^ | Coconino | AZ | 36.198026, -112.051596 |
| Flagstaff^b^ | Coconino | AZ | 35.11667, -111.667 |
| West Okanagan Lake^[39]a^ |  | BC | 50.20889, -119.477 |
| East Okanagan Lake^[39]a^ |  | BC | 49.88792, -119.325 |
| Tallac Historical Site, Lake Tahoe Management Unit^[78-80]a,c^ | El Dorado | CA | 38.93939167, -120.04805556 |
| Echo Summit, Lake Tahoe Basin Management Unit^[13, 79]c^ | El Dorado | CA | 38.81296389, -120.03000000 |
| Meeks Bay, Lake Tahoe^[13]^ | El Dorado | CA | 39.03777, -120.13 |
| Azalea Campground^b^ | Fresno | CA | 36.73877778, -118.96416667 |
| Camp Edison at Shaver lake, Sierra National Forest ^[79]c^ | Fresno | CA | 37.12637500, -119.31472222 |
| Huntington Lake^[78]a,c,d^ | Fresno | CA | 37.24992500, -119.18611111  37.231614, -119.235767 |
| Billy Creek Campground^[78]c^ | Fresno | CA | 37.239122, -119.228311 |
| Crooked Creek Station, Inyo National Forest^[80]c^ | Inyo | CA | 37.58426667, -118.23638889  37.49974, -118.171546 |
| Lake Sabrina Campground, Inyo National Forest^[81]c^ | Inyo | CA | 37.21193889, -118.61388889  37.200, -118.600 |
| Swall Meadows, Inyo National Forest^[80, 81]c^ | Inyo | CA | 37.51138333, -118.63916667 |
| Lee Vining Ranger Station, Inyo National Forest^[78, 80, 81]c^ | Inyo | CA | 37.93831111, -119.12638889 |
| Four Jeffery Campground, Inyo National Forest^[80, 81]c^ | Inyo | CA | 37.23333, -118.566667  37.248577, -118.570508 |
| Pine Glade, Inyo National Forest^[80]c^ | Inyo | CA | 37.57446944, -118.67861111 |
| Manzanar National Historic Site^[80]^ | Inyo | CA | 36.72827222, -118.15444444 |
| Crestview Fire Station, Inyo National Forest^[78, 79, 81-83]a,c^ | Inyo | CA | 37.72255556, -119.02861111 |
| Eagle Lake Field Station^[84]a,c^ | Lassen | CA | 40.55380556, -120.76305556 |
| Mt. Wilson Observatory^[80, 85]a^ | Los Angeles | CA | 34.22583611, -118.0569444  34.22589, -118.057844 |
| Hodgdon Meadow, Yosemite National Park^[79]c^ | Mariposa | CA | 37.79866111, -119.86666667 |
| Wawona, Yosemite National Park^[79]c^ | Mariposa | CA | 37.53689444, -119.65611111 |
| Yosemite National Park^[79]c^ | Mariposa | CA | 37.74172222, -119.57722222 |
| Blue Lake, Modoc National Forest^[80]b,c^ | Modoc | CA | 38.61495278, -119.92500000  41.14265, -120.281  41.1415, -120.281833 |
| Krakatoa Island, Mono Lake^[40]a^ | Mono | CA | 38.0281, -119.041 |
| Mammoth, Twin Lakes^b^ | Mono | CA | 37.61195556, -119.01000000  38.1717, -119.328 |
| Woods Lodge, Inyo National Forest^[79]c^ | Mono | CA | 37.60410556, -119.01305556 |
| Polaris, Truckee River^[86]^ | Nevada | CA | 39.34995556, -120.10055556 |
| Boca Ridge^b,c^ | Nevada | CA | 39.414, -120.01122 |
| Alpine Meadows, West Lake Tahoe^[87]^ | Placer | CA | 39.17618, -120.23 |
| Carnelian Bay, Tahoe^b^ | Placer | CA | 39.22685000, -120.08166667 |
| West Lake Tahoe^b^ | Placer | CA | 39.11667, -120.15 |
| Lake Almanor Campground, Lassen National Forest^[80]^ | Plumas | CA | 40.21792778, -121.17750000 |
| Fern Basin Campground, San Bernardino National Forest^[80]^ | Riverside | CA | 33.78878056, -116.73861111 |
| Marion Mountain Campground, San Bernardino National Forest^[80]^ | Riverside | CA | 33.79189444, -116.73166667 |
| El Prado Group Campground, Cleveland National Forest^[80]^ | San Benito | CA | 32.88837500, -116.45000000 |
| Big Bear, San Bernardino National Forest^[78]a,b,c^ | San Bernardino | CA | 34.26350000, -116.90166667  34.272717, -116.93288 |
| Big Bear Lake, South shore^b^ | San Bernardino | CA | -32.25, -116.9 |
| Holocomb Valley Campground, San Bernardino National Forest^[80, 81]c^ | San Bernardino | CA | 34.30297778, -116.89722222  34.303021, -116.897936 |
| San Gorgonio Campground, San Bernardino National Forest^[80]^ | San Bernardino | CA | 34.17444167, -116.86694444 |
| Laguna/El Prado Campground, Cleveland National Forest^[80]c^ | San Diego | CA | 32.887996, -116.450158  32.88837500, -116.45000000 |
| Manzanita Lake Campground, Lassen Volcanic National Park^[80]c^ | Shasta | CA | 40.53651944, -121.56138889  40.53907, -121.564 |
| Packer Lake Lodge^[72]c^ | Sierra | CA | 39.62175833, -120.65694444 |
| Berger Creek Campground^b^ | Sierra | CA | 39.6278, -120.6451 |
| Sierra City^[12]^ | Sierra | CA | 39.56667, -120.65 |
| Medicine Lake, Modoc National Forest^[80]^ | Siskiyou | CA | 41.58558333, -121.58888889 |
| Juanita Lake Campground, Klamath National Forest^[80]^ | Siskiyou | CA | 41.82240278, -122.12472222 |
| McCloud Ranger Station, Shasta-Trinity National Forest^[79]c^ | Siskiyou | CA | 41.25688889, -122.12527778 |
| Mt. Hebron^[87]^ | Stevens | CA | 41.764, -122.11 |
| Strawberry Lake^14^ | Tuolumne | CA | 39.414, -120.01122 |
| Tuolumne Meadows, Yosemite National Park^[79]c^ | Tuolumne | CA | 37.87148889, -119.36083333 |
| Estes Park^a,e^ | Larimer | CO | 40.377, -105.522  40.251, -105.390 |
| Great Sand Dunes National Park^e^ | Alamosa | CO | 37.901, -105.701  37.880069, -105.620158 |
| West Lake Coeur d'Alene^[13]^ | Kootenai | ID | 47.573661, -116.880304 |
| Spirit Lake^[13]^ | Kootenai | ID | 47.947184, -116.890784 |
| East Lake Coeur d'Alene^[13]^ | Kootenai | ID | 47.565801, -116.72736 |
| Bonner's Ferry^[13]^ | Boundary | ID | 48.797715, -116.279137 |
| South Wild Horse Island, Flathead Lake^[13]a^ | Lake | MT | 47.833053, -114.210964 |
| North Wild Horse Island, Flathead Lake^[14]^ | Lake | MT | 47.851876, -114.207099 |
| Yellow Bay, Flathead Lake^[14]a^ | Lake | MT | 47.878347, -114.032996 |
| East of Yellow Bay, Flathead Lake^[14]a^ | Lake | MT | 47.883794, -114.013129 |
| Melita Island, Flathead Lake^[14]a^ | Lake | MT | 47.820844, -114235222 |
| Big Arm^[14]^ | Lake | MT | 47.801341, -114.222177 |
| Bitterroot Valley^[88]a^ | Ravalli | MT | 46.30497, -114.03176 |
| Lake Como^[88]^ | Ravalli | MT | 46.05487, -114.2413 |
| Hughes Creek^[88]a^ | Ravalli | MT | 45.6114, -114.28916 |
| Mount Charleston | Clark | NV | 36.3, -115.6 |
| Santa Rosa Mountains^b^ | Humboldt | NV | 41.65166667, -117.70750000 |
| Incline Village, West Lake Tahoe | Washoe | NV | 39.246128, -119.952658 |
| Little Valley^b^ | Washoe | NV | 39.276864, -119.824822 |
| Galena^b^ | Washoe | NV | 39.26667, -119.817 |
| Cordova^e^ | Rio Arriba | NM | 36.0, -105.9 |
| Sheep Springs^[15]a^ | San Juan | NM | 36.1, -108.8 |
| East of Salt Lake City^[13]^ | Duchesne | UT | 40.378, -110.467 |
| Badger Mountain^[13]a^ | Douglas | WA | 47.60218, -120.158 |
| Winthrop^[13]^ | Okanogan | WA | 48.460937, -120.26 |
| Bonner Co., Idaho^[13]^ | Bonner | ID | 48.268681, -116.930441 |
| Browne Mountain ^[41]a^ | Spokane | WA | 47.604424, -117.328497 |
| Loon Lake^[13]^ | Stevens | WA | 48.05011, -117.641143 |
| Colville^[13]^ | Stevens | WA | 48.528724, -117.944925 |

^a^ Indicates where *O. hermsi* has also been documented; ^b^ N. Nieto and M. Teglas, *personal communication*; ^c^ Indicates presence of seropositive and PCR positive rodents; ^d^ T. Schwan, *this study*; ^e^ K. Gage, Centers for Disease Control and Prevention, *personal communication*

**S1 References**

78. California Department of Public Health. Vector-borne disease section annual report 2006. Available from: sandiegohealth.org/disease/dhs/vbd_ca_annual_2006.pdf

79. California Department of Public Health. Vector-borne disease section annual report 2007. Available from: sandiegohealth.org/disease/dhs/vbd_ca_annual_2007.pdf

80. California Department of Public Health. Vector-borne disease section annual report 2008. Available from: sandiegohealth.org/disease/dhs/vbd_ca_annual_2008.pdf

81. California Department of Public Health. Vector-borne disease section annual report 2005. Available from: sandiegohealth.org/disease/dhs/vbd_ca_annual_2005.pdf

82. California Department of Public Health. Vector-borne disease section annual report 2002. Available from: sandiegohealth.org/disease/dhs/vbd_ca_annual_2002.pdf

83. California Department of Public Health. Vector-borne disease section annual report 2003. Available from: sandiegohealth.org/disease/dhs/vbd_ca_annual_2003.pdf

84. Cleary M, Theis J. Identification of a novel strain of *Borrelia hermsii* in a previously undescribed northern California focus. Am J Trop Med Hyg. 1999;60(6):883-7.

85. Schwan TG. Tick-borne relapsing fever and *Borrelia hermsii*, Los Angeles County, California, USA. Emerg Infect Dis. 2009;15(7):1026-31.

86. Briggs LH. Relapsing fever in California. J Amer Med Assoc. 1922;79(12):941-4.

87. Fritz CL, Bronson LR, Smith CR, Schriefer ME, Tucker JR, Schwan TG. Isolation and characterization of *Borrelia hermsii* associated with two foci of tick-borne relapsing fever in California. J Clin Microbiol. 2004;42(3):1123-8.

88. Christensen J, Fischer RJ, McCoy BN, Raffel SJ, Schwan TG. Tickborne relapsing fever, Bitterroot Valley, Montana, USA. Emerg Infect Dis. 2015;21(2):217-23.
